# Thresholds of sea‐level rise rate and sea‐level rise acceleration rate in a vulnerable coastal wetland

**DOI:** 10.1002/ece3.3550

**Published:** 2017-11-12

**Authors:** Wei Wu, Patrick Biber, Matthew Bethel

**Affiliations:** ^1^ Division of Coastal Sciences School of Ocean Science and Technology The University of Southern Mississippi Ocean Springs MS USA; ^2^ Louisiana Sea Grant College Program Louisiana State University Baton Rouge LA USA

**Keywords:** ecological thresholds, landscape metrics, northern Gulf of Mexico, sea‐level rise, sea‐level rise acceleration

## Abstract

Feedbacks among inundation, sediment trapping, and vegetation productivity help maintain coastal wetlands facing sea‐level rise (SLR). However, when the SLR rate exceeds a threshold, coastal wetlands can collapse. Understanding the threshold helps address key challenges in ecology—nonlinear response of ecosystems to environmental change, promotes communication between ecologists and resource managers, and facilitates decision‐making in climate change policies. We studied the threshold of SLR rate and developed a new threshold of SLR acceleration rate on sustainability of coastal wetlands as SLR is likely to accelerate due to enhanced anthropogenic forces. Deriving these two thresholds depends on the temporal scale, the interaction of SLR with other environmental factors, and landscape metrics, which have not been fully accounted for before this study. We chose a representative marine‐dominated estuary in the northern Gulf of Mexico, Grand Bay in Mississippi, to test the concept of SLR thresholds. We developed a mechanistic model to simulate wetland change and then derived the SLR thresholds for Grand Bay. The model results show that the threshold of SLR rate in Grand Bay is 11.9 mm/year for 2050, and it drops to 8.4 mm/year for 2100 using total wetland area as a landscape metric. The corresponding SLR acceleration rate thresholds are 3.02 × 10^−4^ m/year^2^ and 9.62 × 10^−5^ m/year^2^ for 2050 and 2100, respectively. The newly developed SLR acceleration rate threshold can help quantify the temporal lag before the rapid decline in wetland area becomes evident after the SLR rate threshold is exceeded, and cumulative SLR a wetland can adapt to under the SLR acceleration scenarios. Based on the thresholds, SLR that will adversely impact the coastal wetlands in Grand Bay by 2100 will fall within the likely range of SLR under a high warming scenario (RCP8.5), highlighting the need to avoid RCP8.5 to preserve these marshes.

## 1 INTRODUCTION

Coastal wetlands are disappearing at an alarming rate in many parts of the world, along with their associated ecosystem services, including carbon sequestration, water quality improvement, flood control, protection from storms, habitat, fishery, recreational opportunities, and cultural values (Costanza et al., 1997; Engle, 2011). Sea‐level rise (SLR), due to ocean thermal expansion, mass loss from glaciers and ice sheets, groundwater extraction, and reservoir impoundment (Gregory et al., 2013), is one of the increasingly important drivers for loss of coastal wetlands in many parts of the world. Concerns arise that SLR is accelerating and will continue to accelerate into the future given the estimated increase in glacial and ice sheet melting and rising concentrations of greenhouse gases (Fasullo, Nerem, & Hamlington, 2016), potentially posing significant threats to coastal ecosystems and human communities (Haigh et al., 2014). Supporting this concern, there is evidence of an increase in the rate of SLR of up to 0.25 mm/year^2^ in the global mean sea‐level (GMSL) data and average sea‐level time series data, after the data were corrected for internal variability for the 20th century and early part of the 21st century (Haigh et al., 2014). Nevertheless, there still exists some debate on whether there is an acceleration in the SLR rate, mainly due to considerable variability and relatively short temporal coverage in sea‐level records (Fasullo, Nerem, & Hamlington, 2016; Gregory et al., 2013).

**Figure 1:**
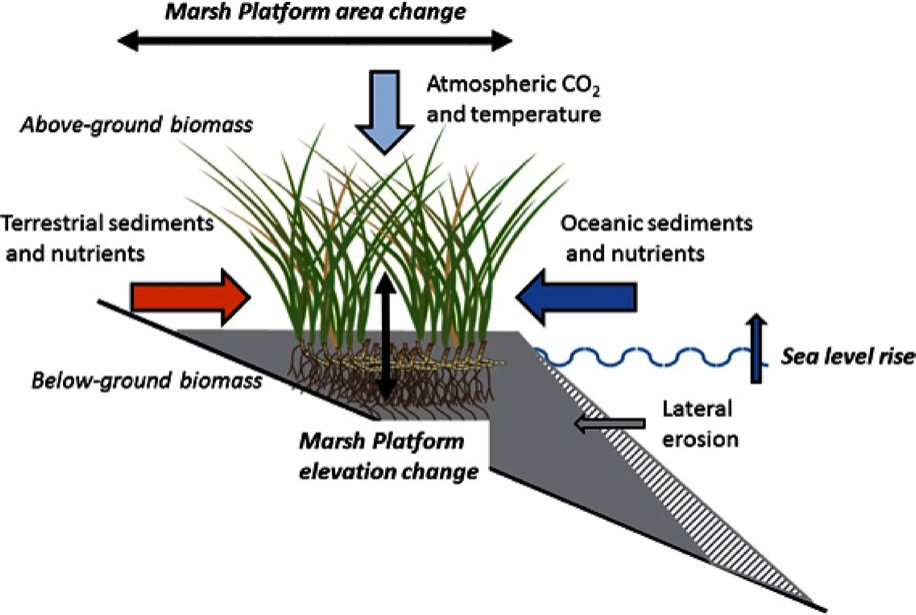
Conceptual model indicating model elements simulated that affect marsh platform elevation and area in response to sea‐level rise (SLR) and climate change drivers. Sustainable marsh platforms require plant biomass and sediment deposited from water column to maintain a positive increase in elevation that meets or exceeds the rates of SLR

Stability of the coastal wetland platform under SLR relies on the balance between inputs due to allochthonous matter deposition and in situ vegetation production, versus losses through subsidence, erosion, and organic matter decomposition (Neubauer, 2008) (Figure 1). It is important to consider relative SLR (SLR + subsidence) when coastal subsidence is substantial. The coastal wetland platform can keep up with low‐to‐moderate SLR (up to 12 mm/year in historical record) due to the feedbacks among inundation, sediment trapping, and vegetation productivity (Jankowski, Törnqvist, & Fernandes, 2017; Kirwan & Guntenspergen, 2009; Kirwan, Temmerman, Skeehan, Guntenspergen, & Faghe, 2016; Morris, Sundareshwar, Nietch, Kjerfve, & Cahoon, 2002). However, when the SLR rate exceeds a threshold beyond which this feedback can no longer be sustained, then the wetland platform can rapidly become tidal or subtidal flats or disappear underwater (Fagherazzi, Carniello, D'Alpaos, & Defina, 2006; Kirwan et al., 2010; Wang & Temmerman, 2013). Kirwan and Megonigal (2013) show that salt marsh habitats could potentially remain viable with a local SLR rate threshold of 7–12 mm/year over geological time when there was no or negligible anthropogenic influence. For reference, during the Holocene postglaciation between 12K and 7K years before present, average rates of SLR were approximately 15 mm/year (Smith, Harrison, Firth, & Jordan, 2011).

In contrast, during the present day with intense human activities, changes in each of the dynamic components involved in balancing wetland platform elevation become accelerated, which may lead to reduced SLR rate thresholds for coastal wetlands. These human impacts include (1) reduced sediment inputs through damming and channelization (Day, Pont, Hensel, & Ibanez, 1995), (2) increased nutrient inputs that likely lower the production of extensive roots and therefore destabilize salt marsh platforms (Darby & Turner, 2008), (3) increased atmospheric CO_2_ concentration that acts as a photosynthesis stimulant to increase vegetation productivity (Cherry, McKee, & Grace, 2009; Langley, McKee, Cahoon, Cherry, & Megonigal, 2009), (4) increased temperature that could simultaneously increase and decrease salt marsh sustainability by concurrently promoting primary productivity but also stimulating decomposition (Kirwan, Guntenspergen, & Langley, 2014; Wu, Huang, Biber, & Bethel, 2017), and (5) accelerated relative SLR that affects vegetation productivity (Morris et al., 2002) and causes more rapid edge erosion resulting in a potential decrease in marsh area. These variables act together to interrupt the balance for wetland platform maintenance. Once the imbalance reaches some tipping point, the total area of coastal wetlands starts to decline rapidly to a new and less desirable state, usually shallow estuarine waters, with very little capability of the wetlands to recover, the classic pattern associated with an ecological threshold state shift.

An ecological threshold can be defined as the value for an environmental driver, beyond which, an abrupt change in ecosystem state, quality, property, or phenomenon will happen, or where small changes in the environmental driver produce large responses in the ecosystem (Groffman et al., 2006). When an ecosystem crosses an ecological threshold, the change is often irreversible, resulting in an alternate stable state; such threshold changes may also exhibit hysteresis behavior that further inhibits return to the original ecosystem condition (Andersen, Carstensen, Hernández‐García, & Duarte, 2009). Thus the concept of ecological thresholds can be useful in implementing proactive policy decisions, for example, deriving critical loads for atmospheric acidic deposition (Burns, Blett, Haeuber, & Pardo, 2008; Porter, Blett, Potter, & Huber, 2005). Environmental management that avoids crossing such a threshold could prevent severe negative consequences on the natural ecosystem, and by extension, human society that depends on it.

The identification of ecological thresholds remains difficult as they are complicated by the nonlinear responses of ecosystems to multiple environmental drivers operating together over multiple spatiotemporal scales. This complexity and nonlinearity can be best captured by a dynamic model that integrates the key components and interactions of ecological factors and processes in ecosystems. Therefore, dynamic modeling, applied to a specific system in detail, coupled with more general and conceptual research, is essential to bridging the gap between theory and application (Groffman et al., 2006).

In this paper, we explore the mechanisms that may cause conversion of wetland habitat to estuarine water through loss of elevation and potential ecological thresholds of SLR on coastal wetlands using a dynamic modeling approach. As a case study, we use a marine‐dominated, sediment‐starved former deltaic marsh system, which is considered to be extremely vulnerable to SLR (Jankowski, Törnqvist, & Fernandes, 2017). There are two complementary ecological thresholds we define in this case study to explore the potential future implications of a state shift. First, the threshold of SLR rate is defined here as the constant value of SLR rate for the entire study period (i.e., a linear increase in sea level from 1988 to 2100), beyond which the coastal wetlands will shift to an irreversible and less desirable state by converting to open water. Second, when the SLR rate is not constant but increases over time (i.e., a nonlinear increase in sea level), a more realistic SLR scenario, we additionally need to consider the threshold of the SLR acceleration rate, defined as the value of changing rate of SLR rate (i.e., second derivative of sea level) beyond which the coastal wetlands will experience a state shift. Under the scenario of an increasing SLR rate, there is a temporal lag before the rapid decline in wetland area becomes evident after the SLR rate threshold is exceeded. The newly developed concept of a SLR acceleration rate threshold can help to quantify this temporal lag. The lagging effects of coastal wetlands’ response to SLR were recognized in the previous studies (e.g., Kirwan & Temmerman, 2009).

The goal of this case study was to focus on a marsh complex that exhibits characteristics making it more vulnerable than many other coastal areas to loss resulting from accelerated SLR. Using a mechanistic model of this specific marsh complex further allowed exploration of the relative contributions from the various processes that occur during accelerated SLR, including potential feedback from variables that are not always considered when studying this question. Our specific hypotheses are as follows:

1. The SLR thresholds will be different if the target year, the environmental factors considered, and landscape metrics used are different.
2. The temporal lag before the rapid decline in wetland area becomes evident after the SLR rate threshold is exceeded will be shortened with higher acceleration rate of SLR.
3. The cumulative SLR a coastal wetland can adapt to is less with higher acceleration rate of SLR.

## 2 MATERIALS AND METHODS

**Figure 2:**
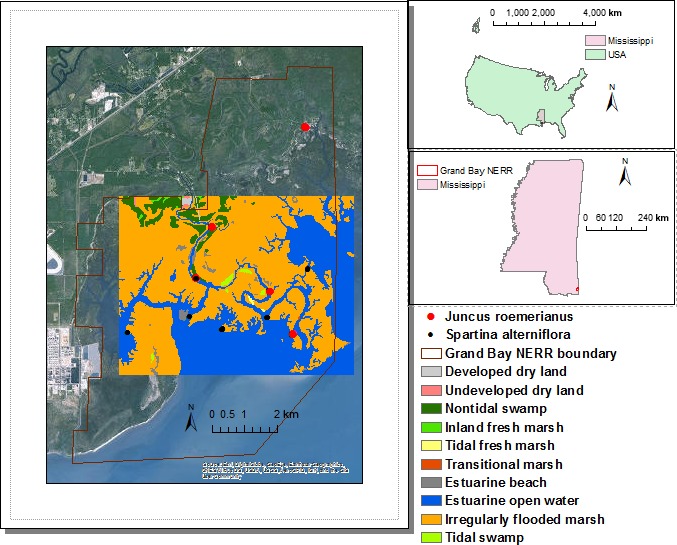
The study area (rectangle in the middle of left panel), mainly in the middle of Grand Bay NERR, with the sampling locations for above‐ground biomass and 1988 NWI map. The inset on the upper left shows the detailed sampling method for a location. The Grand Bay NERR is located in Southeastern Mississippi in Southeastern US (the US map is from the CENSUS, the Mississippi state map is from the Mississippi Automatic Resource Information System, the reserve boundary is from the NERR centralized data management system, the background for the map on the left panel comes from Environmental Systems and Research Institute, and the legend of land cover is for the study area only)

We developed a mechanistic model to predict the impact of SLR on the spatial distribution of coastal wetlands. The mechanistic model incorporated hydrodynamic, geomorphological, and ecological processes, important drivers for elevation change at wetland platform, and therefore wetland change (Figure 1). We applied this model to a case study selecting a microtidal estuarine area with limited upland freshwater and sediment input in the northern Gulf of Mexico, Grand Bay National Estuarine Reserve (NERR) (30°25′47.3″N, 88°25′39.8″W; Figure 2). This study area represents conditions indicative of highly vulnerable coastal wetlands under SLR, allowing us to test the concept of ecological thresholds using a relative short time domain of about 100 years. Due to the approach of mechanistic modeling and representative marine‐dominated system selected, the application of the threshold concept is readily transferrable to other coastal wetlands in marine‐dominated systems where the key input data described in this work are available.

The Grand Bay NERR is located in southeastern Mississippi, with an area of about 3,000 ha of extensive salt marshes dominated largely (>90%) by *Juncus roemerianus* with a small area of *Spartina alterniflora* at the fringe of marshes. Adjacent to the salt marshes is a shallow estuarine area of about 2,800 ha and an average water depth of 0.6–0.9 m influenced by diurnal astronomical tides with an annual average range of about 0.6 m and a maximum range during the summer months of 0.6–0.9 m. The climate is subtropical with hot and humid summers and mild winter conditions (Peterson, Waggy, & Woodrey, 2007).

### 2.1 Model description

The mechanistic model was adapted from both the Marsh Equilibrium Model (MEM) (Morris et al., 2002) for simulating accretion rates and a simplified hydrodynamic model (Dean & Dalrymple, 1991; Fagherazzi & Furbish, 2001; Friedrichs & Aubrey, 1996; Kirwan & Murray, 2008).

#### 2.1.1 Accretion rate

In the modified MEM, we simulated accretion rates by combining sediment settling, sediment trapping by above‐ground biomass, and organic contribution from production of below‐ground biomass. We estimated above‐ground biomass of marsh vegetation based on elevation using a quadratic function derived from our field data for *S. alterniflora* and *J. roemerianus*. We estimated below‐ground biomass using an average ratio of below‐ground biomass to above‐ground biomass across sites with *J. roemerianus* which was 4.6 in this species (>90% coverage in the study area). Then we derived a sediment accretion rate (Equation (1)):

(1) $$\text{acc}=\left(k_{1}S+k_{2}B_{a}\right)h+k_{3}B_{b}/\rho_{\mathrm{bulk}}$$

Where acc denotes accretion rate, h denotes the depth below mean high water; *k*_1_ and *s*_2_ denote the parameters used to estimate the contribution of suspended sediments in the water column to the platform of coastal wetlands, including settling due to gravity and trapping by above‐ground biomass. The sediments in the water columns may represent resuspension of sediments from marsh platforms in the upper estuary or are marine‐derived due to tropical cyclones. Although they do not necessarily represent new sediment inputs in this marine‐dominated and sediment‐deprived wetlands, the exclusion of sediments in the water columns will likely lead to overestimate of coastal wetland loss. The parameter *k*_3_ denotes the effect of root production on sediment accretion via organic matter contribution to the wetland platform as the refractory portion of dead roots is buried. The parameters are calibrated in order for the estimated accretion rate to be close to the measured mean accretion rate at the Grand Bay NERR using the feldspar marker horizon technique on established SET arrays (Cahoon & Turner, 1989; Raposa et al., 2016) and maximize the similarity between the simulated wetland and the map available from the National Wetlands Inventory (NWI) for 2007. B_a_ and B_b_ denote above‐ground (see “Biomass” section) and below‐ground biomass, respectively. S denotes suspended sediment concentration, estimated as a function of latitude based on the field data at our study area (S = 12.22 + 0.002307*latitude), and ρ_bulk_ denotes bulk density which was estimated to be 716.7 kg/m^3^ (Cripps, 2009).

#### 2.1.2 Erosion rate

We simulated the erosion rate using a simplified hydrodynamic model (Dean & Dalrymple, 1991; Fagherazzi & Furbish, 2001; Friedrichs & Aubrey, 1996; Kirwan & Murray, 2008). We modeled an extreme hurricane's impact (e.g., Hurricane Katrina in 2005 which severely affected the area) by increasing the velocity by 10 times, similar to the effect of increasing the maximum wave height to Katrina storm surge height (up to 6 m, https://www.wunderground.com/education/Katrinas_surge_contents.asp, last accessed on 16 April 2017). The erosion rate increased as a result. Less intense storms that have impacted this site have a much reduced effect on this variable and were therefore not included in the simulation.

#### 2.1.3 Elevation

Based on accretion rate, erosion rate, and SLR, we updated the elevation for the marsh platform at each year timestep (*t*) using Equation (2):

(2) $${\text{elv}}_{t}={\text{elv}}_{t-1}-{\text{slr}}_{t}-{\text{sub}}_{t}+{\text{acc}}_{t}-{\text{ero}}_{t}$$

where elv denotes elevation, slr denotes sea‐level rise rate, and sub denotes subsidence rate (negligible at our study area, therefore, we used SLR rather than relative SLR). The vertical datum is mean sea level.

#### 2.1.4 Habitat switch—converting coastal wetland to water

With the elevation updated at each timestep, we predicted whether wetland habitat would be kept or converted into open water. We did not consider tidal or subtidal flats as an intermediate land feature before wetlands are converted into open water, as they are not a year‐round permanent feature in our study area. The unvegetated flats are mostly under water in the summer when tidal amplitude is high and wind is strong. Furthermore, tidal or subtidal flats are not in the National Wetland Inventory data for our study area. We found the lower 2.5% quantile of elevation for salt marshes in our study area to be very close to mean low water (−0.197 m using MSL as datum), so we used mean low water as the lower elevation limit of salt marsh. We assumed the salt marshes were converted to open water if the elevation was lower than this lower limit. The mean low water elevation below which features will become open water is also used in the Sea Level Affecting Marshes Model (SLAMM, http://warrenpinnacle.com/prof/SLAMM/, last accessed on 24 August 2017).

### 2.2 Model inputs

#### 2.2.1 Initial elevation map

We used LiDAR‐derived elevation data collected by the U.S. Army Corps of Engineers acquired in September to October 2005 available at the NOAA Coastal Services Center (https://coast.noaa.gov/dataviewer, last accessed on 25 February 2017). This dataset had a spatial resolution of 2 m and the best vertical accuracy of 7.6 cm in this region and used the datum of NAVD88. In order to keep the same datum, we converted the elevation from NAVD88 to elevation using mean sea level as the new datum by removing the difference between the two datum. The difference is 0.065 m at the Grand Bay NERR, that is, the elevation of mean sea level is +0.065 m in NAVD88 (https://tidesandcurrents.noaa.gov/datums.html?units=1&epoch=0&id=8740166&name=Grand+Bay+NERR%2C+Mississippi+Sound&state=MS, last accessed on 21 January 2017).

#### 2.2.2 Wetland maps

We applied two wetland maps available from the NWI data: the first map from 1988 that we based on to start simulating wetland change (Shirley & Battaglia, 2006), and the second map from 2007 that we used to assess the accuracy of the model simulation for 2007 (https://www.fws.gov/wetlands/data/data-download.html). We converted the polygon files to raster files with a cell size of 2 by 2 meters, consistent with the spatial resolution of the LiDAR elevation map.

#### 2.2.3 Above‐ground biomass

The measured green biomass at the Grand Bay NERR ranged from 204 to 816 g/m^2^ and from 600 to 2044 g/m^2^ for *S. alterniflora* and *J. roemerianus,* respectively (see Supporting Information for sampling method). Based on Morris et al. (2002), there exists an optimum elevation for vegetation productivity. Therefore, we developed a quadratic function to estimate biomass based on elevation. We focused on the area below the salt marshes’ upper limit, which is 0.05 m above mean high water MHW (McKee & Patrick, 1988). As we implemented a nested experimental design (see Supporting Information), we applied a mixed‐effects modeling approach. We chose the best model through model selection method (Burnham & Anderson, 2004) (Table S1), which contained the random effect of sites nested within species (Equation (3)):

(3) $$B_{a.t}=864.28-1022.88\times {\left({\text{elv}}_{t}+0.065\right)}^{2}+\left(1|\text{species}/\text{sites}\right)$$

#### 2.2.4 Validating the model

We compared the vegetation biomass, accretion rate, and velocity at the mud bed simulated from our model to the measured data and other models’ simulations (Braswell, 2010; Passeri et al., 2016; Raposa et al., 2016). We also compared the simulated 2007 wetland distribution from our newly developed model to the NWI data in 2007 (the reference map) using five metrics which accounted for five main components in map comparison: (1) hits: the reference change (land change derived from the 2007 reference map and the initial 1988 map) simulated correctly as change, (2) correct rejections: the reference persistence (land remaining the same from the initial 1988 map to the 2007 reference map) simulated correctly as persistence, (3) wrong hits: reference change simulated incorrectly as change to the wrong category, (4) misses: reference change simulated incorrectly as persistence, and (5) false alarms: reference persistence simulated incorrectly as change (Pontius, Peethambaram, & Castella, 2011). We also calculated the ratio of hits to the sum of hits, misses, false alarms, and wrong hits, also known as “figure of merit,” to quantify how well the model simulated landscape changes (Pontius, Peethambaram, & Castella, 2011).

#### 2.2.5 Deriving thresholds

As the temporal scale is a critical factor affecting resilience of coastal wetlands to SLR, we applied the model to simulate wetland dynamics by 2050 and 2100 under the scenarios of a variety of SLR rates ranging from 4 mm/year (current SLR rate) to 20 mm/year (high end of SLR rate predictions from the IPCC 2013) using an increment of 0.5 mm/year. This provided the predicted total wetland areas under 33 different scenarios of SLR rates, and from this, we derived the thresholds of SLR rate beyond which coastal wetlands will transit to a less desirable state with much smaller emergent wetland areas due to loss of marsh to open water. Note the SLR scenarios are based on global SLR scenarios without considering the local variability (e.g., subsidence variability).

We also applied the model to simulate wetland dynamics under the scenarios of a variety of SLR acceleration rates based on the intermediate‐low, intermediate‐high, and highest SLR scenarios developed from IPCC (IPCC, 2007). As the IPCC only developed the total SLR by 2100 compared to 2000 without any specific SLR predictions in between, a quadratic function was fit to derive the SLR curve to represent an accelerating SLR rate, as previously applied by the National Research Council and the U.S. Army Corps of Engineers and shown in Equation (4) (NRC 1987; Parris et al., 2012):

(4) $$\text{SLR}\left(y\right)=0.0017\times y+b\times y^{2}$$

Where y denotes number of years since 1992 and different values of b represent different scenarios of SLR acceleration. The coefficient b has a value of 1.56 × 10^−4^, 8.71 × 10^−5^, and 2.71 × 10^−5^ for highest SLR, intermediate‐high, and intermediate‐low scenarios. Based on this, we varied b from 2.5 × 10^−5^ to 3.0 × 10^−4^ with an increment of 5.0 × 10^−6^ in the model to represent 56 different acceleration scenarios. For each SLR acceleration scenario, we simulated the resulting spatial distribution of the coastal wetlands by 2050 and 2100. Then we derived the thresholds for acceleration rate based on the total coastal wetland area under different SLR acceleration scenarios.

To identify the ecological threshold of marsh loss, we applied a sigmoidal regression approach (Osland, Enwright, Day, & Doyle, 2013). We fit a sigmoid function to model the relation between total wetland area versus SLR rate or SLR acceleration rate, and then, we determined the inflection point on the fitted sigmoid curve as the threshold. We also explored the thresholds which account for the fertilization effect of the increased concentration of CO_2_ on vegetation productivity, and those based on the landscape metrics other than total area, such as mesh size and mean patch size which could represent landscape fragmentation (Jaeger, 2000; McGarigal, Cushman, & Ene, 2012; Turner & Gardner, 2015).

## 3 RESULTS

Coastal wetlands in the marine‐dominated estuaries, compared to riverine‐dominated estuaries, receive less sediment inputs from upland and represent highly vulnerable wetlands (Jankowski, Törnqvist, & Fernandes, 2017). The SLR thresholds for our chosen system lie in the low ends of sustainability ranges for coastal wetlands in the NGOM.

### 3.1 Model validation results

**Figure 3:**
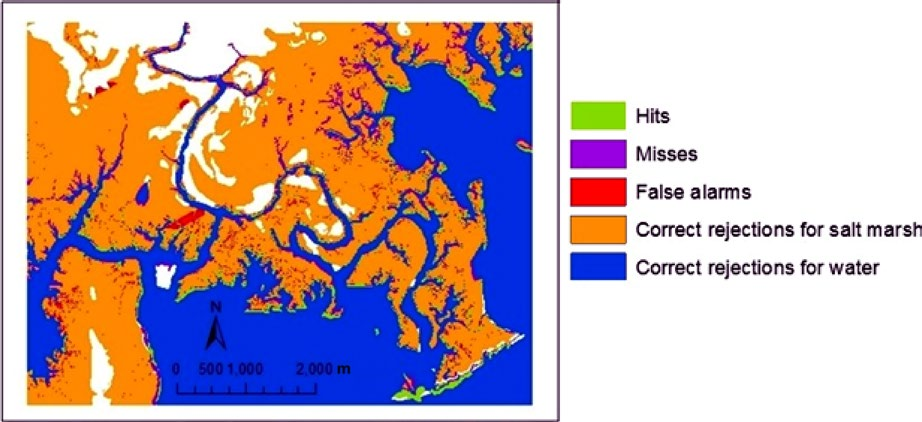
Agreement and disagreement of our 2007 simulations compared to the national wetland inventory (NWI) data of 2007 in reference to 1988 NWI data

Our model simulates wetland change well over ~ 20 years from 1988 to 2007, both in location and amount of change, with a figure of merit of 0.41 (range 0–1). The reference change occupied only 8.1% of the study area with the rest of the area showing persistence of land cover. Land persistence is simulated correctly over 90.4% of the study area. Land change (salt marsh converted to open water) is simulated correctly over 3.9% of the study area (Figure 3). Land change is incorrectly simulated as persistence for 4.2% of the study area, and land persistence is incorrectly simulated as change for 1.5% of area. Overall, the model could correctly simulate 48% of the reference (true) change that occurred between 1988 and 2007 at the Grand Bay NERR, showing a good simulation (Wu, Yeager, Peterson, & Fulford, 2015).

For the time period 1988–2007, the simulated average biomass for the whole study area at the Grand Bay NERR was 808 g/m^2^, consistent with the measurements at the same area in this study and the literature (Braswell, 2010). The simulated average accretion rate (Methods—Equation (1)) for the whole study area was 1.8 mm/year, similar to the average measured data which was 1.4 mm/year using the marker horizon method (Raposa et al., 2016). The simulated average flow velocity, necessary to calculate erosion rate, at the mudflat bed in Grand Bay is ~ 7 m/s in the present day, and it increases to 11 m/s by 2100 with a SLR rate of 4.1 mm/year, very similar to the predicted velocity of 6.1 m/s and 12.2 m/s for the current time and 2100 derived from simulations using the more complex hydrodynamic model ADvanced CIRCulation model (ADCIRC) applied to Grand Bay (Passeri et al., 2016).

### 3.2 Model scenarios and thresholds

**Figure 4:**
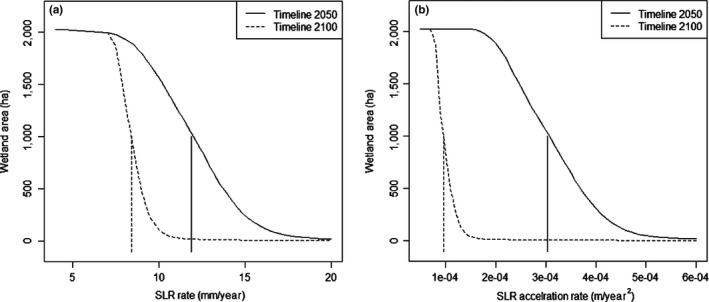
Coastal wetland area for 2050 and 2100 at different sea‐level rise (SLR) rates (a) and different SLR acceleration rates (b). The vertical lines showed the thresholds of SLR rate and SLR acceleration rate for 2050 and 2100, respectively

**Figure 5:**
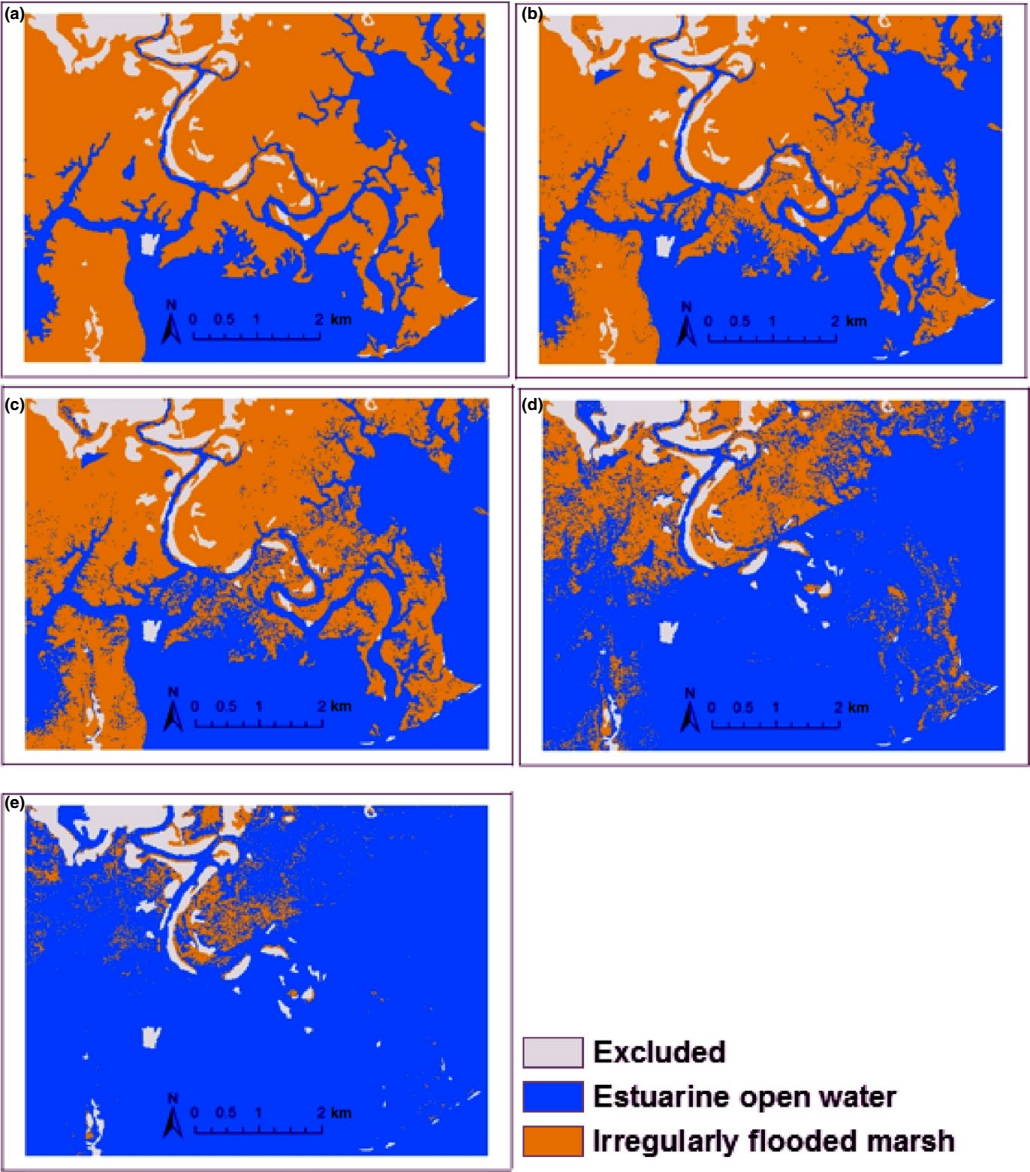
Wetland distribution in (a) 1988, (b) 2100 under the sea‐level rise (SLR) rate of 4 mm/year (current), (c) 2100 under the SLR rate of 7.5 mm/year (~ 1 mm/year lower than the threshold of SLR rate), (d) 2100 under the SLR rate of 8.5 mm/year (~ the threshold of SLR rate), and (e) 2100 under the SLR rate of 9.5 mm/year (~1 mm/year above the threshold of SLR rate)

In the simplistic scenario of a linear SLR rise, as the rate of SLR increases from 4 mm to 7.5 mm/year, the predicted total area of coastal wetlands remaining in the Grand Bay for both 2050 and 2100 decreases marginally (~8% of reduction for 2100) (Figure 4a). The critical SLR rate beyond which the total coastal wetland area in the Grand Bay starts to drop substantially (~56% of reduction for 2100) is ~8.5 mm/year, representing an ecological threshold for this highly vulnerable case study. The decline in coastal wetlands is more gradual for the simulated 2050 area when compared to the later 2100 area. The ecological threshold of SLR rate for 2050 is 11.9 mm/year, whereas this same threshold for 2100 is only 8.4 mm/year. The 2100 SLR rate threshold is comparable to the threshold derived for estuaries with microtide and suspended sediment concentration of ~20 mg/L (average for Grand Bay) in Kirwan et al.'s study (Kirwan et al., 2010). After the SLR rate threshold is exceeded, large portions of the coastal wetlands convert to open water with much smaller total wetland area left, that is, a habitat collapse (Figures 4a and 5). The 2100 threshold of SLR rate of 8.4 mm/year corresponds to a SLR of +0.84 m in 2100 compared to 2000, falling in the likely range of global SLR under RCP8.5^1^ by IPCC AR5 (IPCC 2013), and within both the medium likely range and medium very likely range under RCP 8.5 by Horton, Rahmstorf, Engelhart, and Kemp (2014) (Table 1). However, it is greater than the likely range of global SLR under RCP2.6 by IPCC AR5 IPCC 2013, and the medium likely range and medium very likely range under RCP3 by Horton, Rahmstorf, Engelhart, and Kemp, (2014) (Table 1). These likely or very likely ranges represent the 17th to 83rd percentiles or the 5th to 95th percentiles respectively for estimated future global SLR.

**Table 1:** Sea‐level rise (SLR) predictions from IPCC (2013) and expert survey results in Horton, Rahmstorf, Engelhart, and Kemp (2014)

SLR prediction by 2100 compared to 2000 (m)	IPCC AR5 (2013)		Horton, Rahmstorf, Engelhart, and Kemp (2014)	
	RCP8.5	RCP2.6	RCP8.5	RCP3
Likely range	0.52–0.98	0.28–0.61	0.7–1.2	0.4–0.6
Very likely range	Not available	Not available	0.5–1.5	0.25–0.7

In the more realistic scenario of a nonlinear accelerating SLR curve, as the SLR acceleration rate increases, the threshold of the coefficient b in Equation (4) is 4.81 × 10^−5^ for the 2100 total coastal wetland area, which falls between the intermediate‐low (*b* = 2.71 × 10^−5^) and intermediate‐high emission scenarios (*b* = 8.71 × 10^−5^) (Parris et al., 2012). For 2050, the threshold of *b* is 1.51 × 10^−4^, closest to the b in the highest warming scenario (1.56 × 10^−4^). As the second derivative of the quadratic function in Equation (4) (2 × *b*) represents the changing rate of SLR rise rate (positive numbers representing acceleration), the thresholds of SLR acceleration rate can be calculated as 9.62 × 10^−5^ m/year^2^ and 3.02 × 10^−4^ m/year^2^ for 2100 and 2050, respectively (Figure 4b). Using this SLR acceleration rate threshold number, we derive that SLR will likely rise by a minimum of +0.73 m from 2000 to 2100 (lower than the +0.84 m derived from SLR rate alone), falling within the likely range of SLR by 2100 under the high warming scenario, and slightly exceeding the likely and very likely range under the low warming scenario (Horton, Rahmstorf, Engelhart, & Kemp, 2014; IPCC, 2013). Any SLR larger than 0.73 m from 2000 to 2100 is likely to cause collapse of coastal wetlands in the Grand Bay NERR.

**Figure 6:**
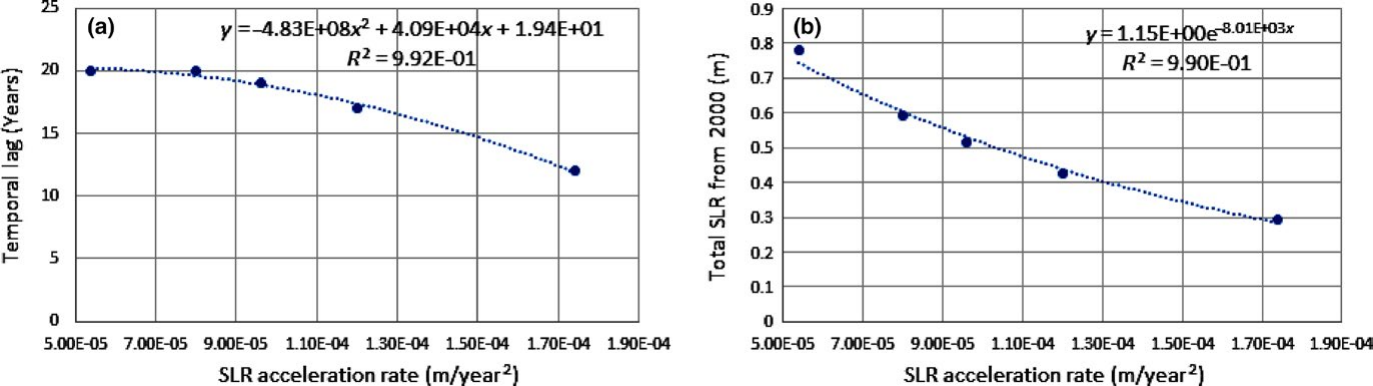
(a) The temporal lag before the total area of coastal wetlands starts to decrease substantially after the threshold of SLR rate is exceeded versus acceleration rate of sea‐level rise (SLR). (b) The total SLR sustained since 2000 that coastal wetlands can take before the total area of coastal wetland starts to decrease substantially versus acceleration rate of SLR. (Note: intermediate‐low scenario has acceleration rate of 5.42 × 10^−5^ m/year^2^, and intermediate‐high emission scenario has acceleration rate of 1.74 × 10^−4^ m/year^2^)

Under this more realistic scenario of SLR accelerating over time, there is a temporal lag before the rapid decline in wetland area in Grand Bay (>400 m^2^ per year) becomes evident after the threshold of SLR rate is exceeded. The newly developed concept of a SLR acceleration rate threshold can help to quantify this temporal lag. The higher the SLR acceleration rate, the quicker coastal wetlands will start to decline significantly after the ecological threshold of SLR rate is exceeded (Figure 6a). If the threshold of the SLR acceleration rate is not exceeded, it takes ~20 years for substantial wetland loss to occur after the ecological threshold of the SLR rate is reached (8.4 mm/year). The delay is much shorter (12–17 years) when the SLR acceleration rate threshold is exceeded in addition to the SLR rate threshold. The total SLR that coastal wetlands can compensate for also depends largely on the magnitude of the accelerated rate of SLR. The higher the SLR acceleration rate, the lower the cumulative sea level the coastal wetland can sustain (Figure 6b).

## 4 DISCUSSION

### 4.1 Processes affecting wetland platform elevation—important role of vegetation biomass

The SLR thresholds below which coastal wetlands are thought to be able to remain resilient in the face of SLR can be up to about 12 mm/year mainly due to the biogenic feedbacks among flooding, sediment trapping, and vegetation growth (Jankowski, Törnqvist, & Fernandes,, 2017; Kirwan & Guntenspergen, 2009; Morris et al., 2002). If this important biological feedback is not accounted for in the model, then the SLR rate threshold for the sediment‐starved coastal marsh we studied reduces from 11.9 mm/year to only 9.5 mm/year for 2050, and it reduces from 8.4 mm/year to only 5.5 mm/year for 2100, which is only 1.4 mm/year higher than the present day SLR rate of 4.1 mm/year in this retrograding deltaic coastal wetland. As the sea level rises, flooding duration becomes longer and more frequent. This promotes sediment settling and trapping. It also helps increase primary productivity at the locations shallower than the depth for optimum primary productivity (Morris et al., 2002) (Equation (3)) and facilitates burial of incompletely decomposed organic matter. All of these mechanisms increase accretion rates on the wetland platform and make wetlands resilient toward increasing SLR, but only up to a threshold condition. After threshold exceedance, it will be harder for coastal wetlands to keep up with the ever‐increasing rates of SLR predicted into the future, even with these biogenic feedback mechanisms.

There are two major processes that affect elevation of the wetland platform: deposition and erosion. We find through the dynamic modeling that deposition reduction in this retrograding delta has a larger impact on future wetland area compared to deposition increase or change in erosion rate. For 2050 at the Grand Bay NERR, the model predicts a 0.181% reduction and a 0.226% increase in coastal wetland area if we increase and decrease erosion rate by 50% respectively under the present day SLR rate of 4.1 mm/year. The model also predicts a 0.266% increase and a 1.75% reduction in coastal wetland area if we increase and reduce deposition rate by 50%, respectively. For 2100, the reduction and increase are predicted to be 0.181% and 0.225% if we increase and decrease erosion by 50%, respectively. The predicted increase and reduction are 0.266% and 3.49% if we increase and decrease deposition by 50%, respectively. The change of erosion and increase in deposition have a similar effect for 2050 and 2100, but the reduction in deposition will have a larger impact on wetland loss further into the future (2100 vs. 2050). This finding is particularly important for this case study where storms may translocate sediments on to the marsh, in essence providing a mechanism to increase deposition within localized areas. We presume that coastal wetlands in less sediment‐starved river delta systems would have better resilience against erosion losses as SLR increases and highlight here that our study system should be considered on the highly vulnerable end of the coastal wetland continuum.

Deposition processes are contributed by both suspended sediments in the water column (either riverine or oceanic derived) and biomass production of vegetation. We find biomass reduction has a larger impact on the total area of coastal wetlands in this retrograding delta compared to increasing biomass or changing sediment concentration in the water column under the current SLR rate. When we increase and decrease sediment concentration in the water column by 50% under the present day SLR rate, the model predicts a 0.0138% increase and a 0.0155% decrease in total wetland area respectively for 2050 (Table 2). The predicted increase and decrease in total wetland area are 0.254% and 1.49% for 2050 if we increase and decrease above‐ and below‐ground biomass by 50% respectively under the current SLR rate. The change of biomass and sediment concentrations have an even larger impact on the total area of coastal wetlands if we consider a higher than present day SLR rate (e.g., 8.4 mm/year) and predicted these impacts further into the future (e.g., 2100) (Table 2). This shows vegetation productivity is a more important factor than suspended sediments to determine the deposition rate in this freshwater‐limited estuary, consistent with the main accretion mechanism in marine‐dominated and sediment‐deprived systems. As such, creating coastal wetlands at an optimum elevation (e.g., 0.065 m below current mean sea level in this region theoretically) to maximize biomass production will likely increase success of restoration efforts and improve resilience of coastal wetlands to SLR.

**Table 2:** The percent change of the total area of coastal wetlands in Grand Bay for 2050 and 2100 under the SLR rate of 8.4 mm/year

Driver	Change in driver (%)	Percent change of total area for 2050 (SLR: 4.1 mm/year)	Percent change of total area for 2100 (SLR: 8.4 mm/year)
Sediment concentration	+50	+0.0138	+11.2
	−50	−0.0155	−9.81
Biomass	+50	+0.254	+107
	−50	−1.49	−84.6

An important component of biomass production is below‐ground biomass. We applied the mean of 4.6 as the ratio of below‐ to above‐ground biomass. If we used the median of 2.4 as the ratio in the model, the SLR thresholds reduce to 11.6 mm/year from 11.9 mm/year for 2050 and to 8.1 mm/year from 8.4 mm/year for 2100.

### 4.2 Additional climate change drivers

Sea‐level rise generally couples with other environmental factors to affect coastal wetlands under climate change (Osland et al., 2016). Elevated temperature has two opposite effects on vegetation. It can promote vegetation productivity but simultaneously can increase decomposition of soil organic matter (Charles & Dukes, 2009; Kirwan & Blum, 2011; Kirwan, Guntenspergen, & Lanley, 2014). While elevated temperatures during the winter season can be beneficial to vegetation productivity, during the summer temperatures in the future may begin to exceed physiological optima resulting in decreased productivity (Hatfield & Prueger, 2015; Schlenker & Roberts, 2009). Similarly, elevated summer temperatures are likely to result in more rapid decomposition of unburied litter (Wu, Huang, Biber, & Bethel, 2017), depriving the marsh of organic matter on the marsh surface. The quicker disappearance of above‐ground plant litter could potentially reduce sediment deposition (Rooth, Stevenson, & Cornwell, 2003), leading to lower accretion rates. The effects of elevated year‐round soil and water temperatures on buried organic matter decomposition are less certain, but have the potential to also reduce the amount of organic matter sequestered (Davidson & Janssens, 2006; Kirwan, Guntenspergen, & Lanley, 2014). Whether temperature has positive or negative impacts on the sustainability of coastal wetlands depends largely on which of these effects will dominate. On the other hand, rising atmospheric CO_2_ concentration can act as fertilizer to promote vegetation productivity and therefore has the potential to increase coastal wetlands’ resilience to SLR (Cherry, McKee, & Grace, 2009; Langley, McKee, Cahoon, Cherry, & Megonigal, 2009; Ratliff, Braswell, & Marani, 2015). When we account for the medium fertilization effect of higher CO_2_ concentration, that is, 39% increase in above‐ground productivity and 33% increase in below‐ground productivity by 2100, we find the threshold of SLR rate increases from 8.4 mm/year to 10.3 mm/year for 2100. The magnitude of increase in the SLR rate threshold accounting for medium fertilization effect of CO_2_ is larger than the results in Ratliff, Braswell, and Marani, (2015), stressing the importance of vegetation productivity on the deposition process and increasing the resilience of coastal wetlands to SLR at the Grand Bay NERR. Even with the higher threshold of SLR rate by the CO_2_ fertilization effect, the accumulated SLR by 2100 still falls within the likely and very likely ranges of SLR under the RCP8.5 scenario, but is much larger than the likely and very likely range of SLR under the low emission scenario (Horton, Rahmstorf, Engelhart, & Kemp, 2014; IPCC, 2013). This indicates that there is a chance that wetland area will experience a significant reduction under the high CO_2_ emission scenario, but this is not as likely under the low CO_2_ emission scenario, when the effect of increasing concentration of CO_2_ is explicitly taken into consideration. These various effects were able to be tested quantitatively using the dynamic model and serve to illustrate the complex interactions and feedback mechanisms that are important to coastal marsh sustainability into the future. Many of these interactions have not been well studied in relation to SLR rate and SLR acceleration thresholds in other systems, and this remains an area of future research needs.

### 4.3 Spatiotemporal implications of saltmarsh landscape

It is critical to account for temporal effects in assessing the resilience of coastal wetlands to SLR and deriving the thresholds of SLR rate and SLR acceleration rate. When our target year changes from 2050 to 2100, both threshold levels decrease substantially, indicating a higher likelihood of marsh habitat collapse in 2100 than 2050. This has important implications for designing climate mitigation and adaption plans. While it seems in this case study that coastal wetlands are resilient to SLR by 2050 under both low and high emission scenarios, it is very likely that highly vulnerable coastal wetlands like Grand Bay could collapse by the end of the century, especially under the high warming scenario.

When we study the thresholds of SLR for coastal wetlands, we generally focus on the total area of coastal wetlands in a region and fail to discuss other landscape metrics which play an important role in describing spatial patterns that are relevant for ecosystem functions. The thresholds of SLR rate in Grand Bay are 11.9 mm/year for 2050 and 8.4 mm/year using total wetland area as a response metric. When we use mesh size, the thresholds become 11.8 mm/year for 2050 and 8.3 mm/year, similar to the values if we use total area as a metric. However, if we use mean patch size to describe the spatial pattern of coastal wetlands, the thresholds of SLR rate decrease to 7.8 mm/year for 2050, and 7.3 mm/year for 2100, smaller than the values derived using total area. Policy makers need to consider the management goal and choose the most appropriate landscape metrics as the basis for deriving ecological thresholds. Total wetland area is a commonly used metric to evaluate restoration success but may not be the most appropriate one to use. For example, if the management goal is related to a fishery, then the ratio of perimeter to area, which reflects both the area and shape of a patch, may be more relevant to habitat use of fish (Meynecke, Shing, Duke, & Warnken, 2007) and therefore should be considered as the base for deriving ecological thresholds.

### 4.4 Complementary SLR thresholds as a tool to communicate with policy makers

It is challenging to communicate the importance of the SLR rate threshold with policy makers, when the value is generally only a few millimeters per year. In this case, the threshold of SLR acceleration rate, and more importantly its implications for the temporal lag before the rapid decline in wetland area becomes evident after the threshold of the SLR rate is exceeded, and the cumulative SLR coastal wetlands can sustain, can be a valuable concept in getting the message of SLR impact across. Framing the wetland loss discussion around number of years (e.g., 17 years) and total SLR (e.g., 60 cm by 2100) can be more useful to coastal managers and policy makers than using the SLR rate or SLR acceleration rate threshold alone.

The demonstrated ecological thresholds of SLR rate and SLR acceleration rate in this case study show the rise of sea level by 2100 falls within the likely range of SLR under the RCP8.5 scenario but is larger than the very likely range of SLR under the RCP 2.6 or 3.0 scenario, therefore, controlling CO_2_ emissions so the thresholds of SLR will not be exceeded becomes important to keep coastal wetlands from collapsing between 2050 and 2100. To keep the total area of coastal wetlands from dropping precipitously, coastal managers and policy makers also need to consider building coastal wetlands at appropriate elevations to maximize vegetation productivity and limiting land development near coastal wetlands to facilitate upland migration of coastal wetlands.

Although these ecological thresholds have been questioned on their appropriate use in natural resource management, the thresholds help address key challenges in ecology—how ecosystems will respond to environmental conditions that do not exist at present or in history and how multiple environmental factors interact to affect ecosystems in a nonlinear way. Knowing SLR thresholds can help determine whether coastal wetlands will persist for the next 100 years. This is critical information to better understand the role that coastal wetlands will play in carbon offset trading programs (Anderson et al., 2016). In addition, the ecological thresholds can guide the derivation of other types of thresholds, such as a utility threshold (indicating where small changes in environmental conditions produce substantial change in management outcomes) and a decision threshold (representing values of a state variable that when exceeded should elicit management action), which are more relevant to resource management goals (Guntenspergen & Gross, 2014; Samhouri, Levin, & Ainsworth, 2010). SLR thresholds are potentially important for policy‐making processes in climate change mitigation and coastal management, to ensure critical ecological thresholds that preserve coastal wetlands are not exceeded.

## 5 CONCLUSIONS

We presented the first study on the threshold of SLR acceleration rate, and the first comprehensive threshold analysis which accounts for the temporal scale, the interaction of SLR with other environmental factors, and landscape metrics used. We tested them in a highly vulnerable and sediment‐starved estuarine system. Based on the total wetland area, the threshold of SLR rate for our retrograding delta study area is 11.9 mm/year for 2050, and it drops to 8.4 mm/year for 2100. The thresholds of SLR acceleration rate are 3.02 × 10^−4^ m/year^2^ and 9.62 × 10^−5^ m/year^2^ for 2050 and 2100, respectively. If we account for the fertilization effect of the increased concentration of CO_2_ on vegetation productivity, the threshold of SLR rate increases to 10.3 mm/year for 2100. If we use mean patch size as the landscape metric, the SLR rate threshold drops to 7.3 mm/year for 2100. Both the thresholds of SLR rate and SLR acceleration rate make future SLR fall within the likely range of SLR by 2100 under the high warming scenario (RCP 8.5) and exceed the very likely range under the low warming scenario (RCP 2.6 or 3.0).

The broader application of this work comes from managers being able to recognize the likelihood of a shorter temporal lag before substantial wetland loss under a higher SLR acceleration rate and better understand what cumulative SLR a coastal wetland can sustain. This will affect the timing or sequencing of effective mitigation planning and allows more confidence in projecting marine‐dominated wetland loss given particular climate change scenarios.

This study illustrates a transferrable and useful method for evaluating coastal wetlands’ nonlinear response to SLR, especially in marine‐dominated systems, and facilitating the enhanced design of mitigation and adaption policy under future climate change projections. The management implications from this case study highlight the need to go beyond simple metrics when evaluating coastal areas that are highly vulnerable to future state transitions in order to implement more‐informed, proactive, and effective practices in mitigating SLR impacts before it is too late.

## CONFLICT OF INTEREST

None declared.

## AUTHORS CONTRIBUTION

WW conceived and designed the research, developed the model, and analyzed the results. PB created the conceptual model figure and contributed to discussion. MB contributed to discussion. WW wrote the manuscript with the contributions from all authors.

## Endnote

1 RCP represents representative concentration pathways, the scenarios adopted by IPCC AR5 (2013). RCP8.5 represents high CO_2_ emission or high warming scenario in which atmospheric CO_2_ concentration will reach 930 ppm and temperature will be 4.5°C above preindustrial temperature, and RCP2.5 or RCP3 represents low CO_2_ emission or low warming scenario in which atmospheric CO_2_ concentration will reach 421 ppm and temperature will be <2°C above preindustrial temperature.
