# Supplementary material for: Thresholds of sea‐level rise rate and sea‐level rise acceleration rate in a vulnerable coastal wetland
Source: Ecol Evol. 2017 Nov 12;7(24):10890–903. doi: 10.1002/ece3.3550 (PMC5743734; doi:10.1002/ece3.3550)
Supplement: Supplementary file 1 [file ECE3-7-10890-s001.docx]

**Supporting Information – Sampling and modeling of aboveground biomass**

We collected peak-season biomass at eleven randomly selected sites within 30 meters of coastlines in the Grand Bay NERR in August 2012, five for *Juncus roemerianus* using a quadrat of 15x15 cm, six for *Spartina alterniflora* using a quadrat of 25x25 cm (Fig. 2). For each sampling site, we selected 5 subsites with one subsite in the middle, four subsites 5 meters away from the middle subsite in the shape of cross (Fig. 2 inset). For each subsite, we collected duplicate samples with a distance of ~1 m from each other. Altogether, we collected 50 biomass samples for *J. roemerianus* and 60 biomass samples for *S. alterniflora*. We cut off all the marsh plants within each sampling quadrat at the ground level and we collected all the leaves above the ground, including the green and dead ones placed into sampling bags, and transported them to the lab within the same day. In the lab, we separated green shoots from the dead ones, cut them, and dried them in the oven at 70 ^o^C for three to four days until constant weight. Then we weighed the dry biomass of green shoots. We averaged the biomass of duplicate samples to get live above-ground biomass at a given subsite.

We compared the aboveground biomass models without random effects, with random effect of site only, with random effect of species only, with random effect of sites within species, with random effect of site and species but no nested structure, and we found the model with random effect of sites within species had the lowest Akaike Information Criterion (AIC) so we chose it to estimate biomass for the whole study area at a spatial resolution of 2 meters (Table S1). AIC, an estimator of the expected Kullback discrepancy between the true model and a fitted candidate model (Eq. S1):

$AIC=-2\log(L(\hat{\theta}|data))+2K$ - Eq. S1

Where $\log(L(\hat{\theta}|data))$ is the maximum log-likelihood ($\hat{\theta}$ denotes estimated model parameters), and K is the number of parameters. The absolute values of AIC are not meaningful, but the relative values and difference of AIC can be used as a guideline for model selection (Burnham and Anderson, 2004). The lower the AIC, the better is the model prediction. A rule of thumb is that two models are essentially undistinguishable if the difference in AIC is smaller than 2.

Table S1 Sets of candidate linear mixed models to predict biomass. The optimum random effect was site nested within species (B denotes biomass in g/m^2^, and E denotes elevation in meters^1^).

| Random effects | Fixed effects | p-value | AIC |
| --- | --- | --- | --- |
| No | B=597.30+387.89×(E+0.065)^2^ | 0.6197 | 518.68 |
| Site | B=702.24-891.58×(E+0.065)^2^ | 0.1313 | 471.85 |
| Species | B=863.22-1013.35×(E+0.065)^2^ | 0.0236 | 478.31 |
| Site + Species | B=863.22-1013.35×(E+0.065)^2^ | 0.0236 | 482.31 |
| Site nested within species | B=864.28-1022.88×(E+0.065)^2^ | 0.0528 | 465.54 |

^1^Note: E has the datum of mean sea level.

Reference

1. Burnham, K.P. & Anderson, R.P. (2004). Multimodel Inference: Understanding AIC and BIC in Model Selection. *Sociol. Methods Res.*, 33, 261–304.
